# Causal inference in multi-state models–sickness absence and work for 1145 participants after work rehabilitation

**DOI:** 10.1186/s12889-015-2408-8

**Published:** 2015-10-23

**Authors:** Jon Michael Gran, Stein Atle Lie, Irene Øyeflaten, Ørnulf Borgan, Odd O. Aalen

**Affiliations:** Oslo Centre for Biostatistics and Epidemiology, Department of Biostatistics, University of Oslo, Oslo, Norway; Department of Clinical Dentistry, University of Bergen, Bergen, Norway; National Centre for Occupational Rehabilitation, Rauland, Norway; Uni Research Health, Bergen, Norway; Department of Mathematics, University of Oslo, Oslo, Norway

**Keywords:** Multi-state models, Causal inference, Sickness absence, Survival analysis, Cohort study, Registry data

## Abstract

**Background:**

Multi-state models, as an extension of traditional models in survival analysis, have proved to be a flexible framework for analysing the transitions between various states of sickness absence and work over time. In this paper we study a cohort of work rehabilitation participants and analyse their subsequent sickness absence using Norwegian registry data on sickness benefits. Our aim is to study how detailed individual covariate information from questionnaires explain differences in sickness absence and work, and to use methods from causal inference to assess the effect of interventions to reduce sickness absence. Examples of the latter are to evaluate the use of partial versus full time sick leave and to estimate the effect of a cooperation agreement on a more inclusive working life.

**Methods:**

Covariate adjusted transition intensities are estimated using Cox proportional hazards and Aalen additive hazards models, while the effect of interventions are assessed using methods of inverse probability weighting and G-computation.

**Results:**

Results from covariate adjusted analyses show great differences in sickness absence and work for patients with assumed high risk and low risk covariate characteristics, for example based on age, type of work, income, health score and type of diagnosis. Causal analyses show small effects of partial versus full time sick leave and a positive effect of having a cooperation agreement, with about 5 percent points higher probability of returning to work.

**Conclusions:**

Detailed covariate information is important for explaining transitions between different states of sickness absence and work, also for patient specific cohorts. Methods for causal inference can provide the needed tools for going from covariate specific estimates to population average effects in multi-state models, and identify causal parameters with a straightforward interpretation based on interventions.

## Background

Data on sickness benefits is a valuable source for analysing sick leaves, disability and employment, but due to the complexity of such data the choice of measurement type and analysis can be challenging [1]. However, recent work using data from Norwegian [2, 3] and Danish registries [4–7] has proved that multi-state models [8–13] can be a very successful framework for analysing this kind of data. For example, when studying the effect of participating in work rehabilitation programs, events such as return to work, onset of sick leave benefits or work assessment allowance can hardly be seen as single time-to-event outcomes, but rather as a set of events which define states that the individuals move between. Multi-state modelling, as an extension of traditional survival analysis, offers a unified approach to the modelling of the transitions between such states.

National registries with data on sickness benefits is a good basis for many types of analyses. The data are typically complete, and detailed information is collected on the type of benefits and dates when they are given. Additional information on the individuals receiving benefits is often available or can be obtained in even greater detail by coupling such registry data with cohort data where detailed information is available.

The assessment of possible interventions with the purpose of reducing sickness absence is an important aim when analysing sickness benefit data, and identifying successful interventions could have a possible large economic impact [14]. In this paper we will focus on two such interventions, which both have received a lot of attention. One is the effect of partial compared to full time sick leave benefits, see e.g. [14–17], and the other is the effect of a cooperation agreement on a more inclusive working life, see e.g. [18]. In the Nordic countries there have been political initiatives for expanded use of partial sick leave. Part-time work may be beneficial, and a feasible way to integrate individuals with reduced work ability in working life, if the alternative is complete absence from work [15, 17]. In Norway, an agreement on more inclusive working life was signed by the Government and the social partners in employers and employees’ organisations in 2001, and was renewed in 2005, 2010 and 2014. One of the main aims of this tripartite agreement has been to reduce the amount of individuals on sick leave and disability pension.

Even though some attempts have been made to conduct randomized trials to assess interventions for reducing sick leave [19, 20], the execution of such experiments is challenging and not very commonly seen. As for using observational data to identify the effect of such interventions, numerous attempts have being made, see e.g. [21–24]. There has also been a massive methodological development over the last decades within the field of causal inference [25–27], providing a formal framework for identifying parameters similar to those in randomized trials from observational data. Such methods can also be employed in a multi-state model setting, but this has hardly been done yet.

Earlier work on multi-state models for Norwegian registry data on sick leave benefits has also been in the form of cohort follow-up studies [2, 3], but without using the detailed covariate information available in these cohorts. In this paper we extend the analysis of Øyeflaten et al. [3], analysing transitions between sick leave benefits, work assessment allowance, disability pension and work for patients participating in work rehabilitation programs. Formally, we make three extensions to the analyses in the original paper. First of all, we cover a larger multi-center cohort, about double in size. Secondly, we utilize the detailed covariate information which is available for this cohort to estimate covariate specific state transition probabilities. Doing this, both proportional hazards and additive hazards models are here being considered for the purpose of estimating the transition intensities. Last but not least, we explore three different approaches based on classical methods from the causal inference literature to estimate the effect of interventions in multi-state models.

The purpose of this paper is therefore twofold; to use multi-state models to study sickness absence and work based on detailed covariate information for a cohort of participants after work rehabilitation, and, to illustrate how methods from the causal inference literature can be used to estimate the effect of interventions in such a multi-state model framework. Detailed covariate information is, of course, central in making covariate specific predictions in a multi-state model, but even more important when estimating the causal effects of interventions from observational data. The statistical and causal assumptions needed will be discussed specifically.

Covariate information has been used in multi-state models before for predicting sick leaves and related outcomes, in two recent papers on Danish data [4, 7]. The main difference between the data in these studies and the data in the present study is that the Danish data cover a much larger cohort, while the Norwegian data include more detailed information on the health of the participants. The latter is important for precise patient predictions and for adjusting for confounding when aiming at drawing causal conclusions. None of the earlier studies consider the estimation of causal effects of interventions in a multi-state setting.

With the increasing attention on multi-state modelling of event-history data, more and more software packages have been made available, especially in R [28]; for example the mstate [29], msm [30] and msSurv [31] packages. See the latter, or the books of Beyersmann et al. [32] and Willekens [33], for detailed overviews of available R packages. The computations in this paper has been performed in R using the surv and mstate packages and by standalone code written by the first author.

## Methods

### Data sources

#### A multi-center cohort

The patients being analyzed are part of a multi-center cohort study with the purpose of studying how health complaints, functional ability and fear avoidance beliefs explain future disability and return-to-work for patients participating in work rehabilitation programs. Data has been collected on 1155 participants from eight different clinics offering comprehensive inpatient work rehabilitation. Mean time on sickness benefits during the last two years before admittance to the work rehabilitation program, were 10 months (SD = 6.7). All participants gave informed consent, allowing for follow-up data on sickness absence benefits to be obtained from national registries, and answered comprehensive questionnaires during their stay at the clinic. The study was approved by the Medical Ethics Committee; Region West in Norway (REK-vest ID 3.2007.178) and the Norwegian social science data services (NSD, ID 16139). The data collected through questionnaires includes various background information together with detailed health variables such as subjective health complaints, physical function, coping and interaction abilities, and fear-avoidance beliefs. See Øyeflaten et al. [34] for more details on the cohort.

#### Data on sickness benefits

All Norwegian employees are entitled to sickness benefits such as sick leave benefits, work assessment allowance or disability benefits, if incapable of working due to disease or injury. The employer pays for the first 16 days of a sick leave period, and thereafter The Norwegian Labour and Welfare Administration (NAV) covers the disbursement. Data on these benefits, both the ones covered by the employer and NAV, was obtained from NAV’s register, which contains information on the start and stop dates of sickness benefits given from 1992 and onward for the entire Norwegian population.

#### Data for current analysis

Out of the original 1155 participants in the multi-center cohort study, we excluded 4 individuals with an unknown date of departure from their rehabilitation center, 1 individual who had not answered the relevant questions on subjective health complaints and 5 individuals already on disability pension at baseline, and were left with a study sample of 1145 participants. Baseline was set to the time of departure, which varied between May 16th 2007 and March 25th 2009. Individuals were followed up with regard to their received sickness benefits until July 1st 2012, which was the date of data extraction from NAV.

### A multi-state model for sickness absence and work

The occurrence of an event in survival analysis can be seen as a transition from one state to another, for example from an alive state to a death state. The hazard rate corresponds to the transition intensity between these two states. Multi-state models form a flexible framework allowing for the standard survival model to be extended by adding more than one transition and more than two states. A detailed introduction to multi-state models can be found in review papers such as Hougaard [8], Commenges [9], Andersen and Keiding [10], Putter et al. [11] and Meira-Machado et al. [12], or the book chapter by Andersen and Pohar Perme [13].

Sickness absence and disability data is a good example of data that are suitable for being modelled within the multi-state framework. Changing between work and being on various types of sickness benefits over time can naturally be perceived as moving between a given set of states.

**Fig. 1:**
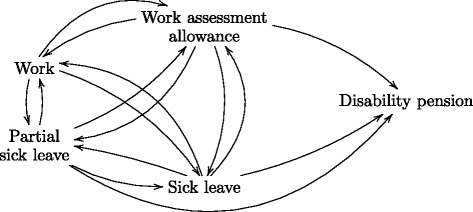
Multi-state model for sickness absence and work. A model for the transitions between work (no registered benefits), sick leave benefits, partial sick leave benefits, work assessment allowance and disability pension, for patients being discharged from clinics offering comprehensive inpatient work rehabilitation

In Norway, employees on partial or full sick leave can be fully compensated through sick leave benefits for up to a year, after which they can be entitled to work assessment allowance. If their underlying health condition provides reasons for it, they may be granted a disability pension or further partial sick leave benefits. The latter is actively recommended by the authorities [35]. Partial sick leave can be graded from 20 to 99 %. Based on these policies we define five states that the participants can move between after being discharged from the rehabilitation centers: work (no received benefits), sick leave, partial sick leave, work assessment allowance and disability pension, and we propose the multi-state model illustrated in Fig. 1. At baseline, when being discharged from the rehabilitation center, individuals can start in any of the first four states.

Individuals are defined as being on sick leave when receiving full sick leave benefits, on partial sick leave when receiving sick leave benefits graded below 100 % and on disability pension when receiving disability pension on unlimited terms. Work assessment allowance is a intermediate benefit typically given between sick leave and disability pension. It is granted for individuals going through medical treatment or rehabilitation, or to others that might benefit from vocational rehabilitation actions. There is an upper limit of four years for receiving work assessment allowance. When individuals do not receive any sickness benefits, they are per definition in work. The only exception is when there are gaps with no benefits before receiving disability pension – as there are no real transitions directly from work to disability, such gaps are attributed to the most recently received benefit. To avoid including non-genuine transitions, benefits with a duration of only one day have been discarded. When there were benefits registered which overlapped in time, the newest registered benefit was used.

As for initial states; 178 patients started in the work state (receiving no benefits) after being discharged from the rehabilitation center, 106 were on partial sick leave benefits, 496 on full sick leave benefits and 365 were on work assessment allowance. Disability pension was defined to be an absorbing state in the multi-state model, as few transitions were observed to go out of this state in the original data. The total number of subsequent transitions between the five states within the study window is shown in Table 1.

**Table 1:** Transition summary. Total number of transitions between the five states work (state 1), partial sick leave (state 2), sick leave (state 3), work assessment allowance (state 4) and disability pension (state 5) based on registry data from The Norwegian Labour and Welfare Administration (NAV) for participants in the multi-center cohort

	From: / To:	1	2	3	4	5
Work	1	0	378	2235	406	0
Partial sick leave	2	732	0	275	106	27
Sick leave	3	2397	614	0	265	66
Work assessment allowance	4	343	48	348	0	183
Disability pension	5	0	0	0	0	0

Covariate information include age at baseline, gender, marital status, whether a cooperation agreement on a more inclusive working life is present, educational level, type of work, income, working ability score when entering rehabilitation and diagnosis group at baseline. All covariates are based on information from the questionnaires, except information on type of diagnosis which is retrieved through the ICPC code when available in NAV’s register, and partly from the cohort data at the time of entering the rehabilitation. The current diagnosis at any given time is defined as the last given diagnosis. Note that these selected covariates only are one out of many possible representations of the information in the original data source, constructed to sufficiently describe the differences between patients. Detailed statistics on the covariates are found in Table 2.

**Table 2:** Descriptive statistics. Description of selected covariates collected from questionnaires to the multi-center cohort and Norwegian Labour and Welfare Administration (NAV) data (n=1145)

Characteristic	Total number (%)
	or mean (sd)
Age	45.7 (9.1)
Gender	
Female	798 (70 %)
Male	347 (30 %)
Marital status	
Not married	345 (30 %)
Married	716 (63 %)
Not answered	84 (7 %)
College or university education	
No	743 (65 %)
Yes	309 (27 %)
Not answered	93 (8 %)
Type of work	
Manual labour	222 (19 %)
Office or administration	174 (15 %)
Educational	142 (12 %)
Health and social services	247 (22 %)
Service work	165 (14 %)
Not answered	195 (17 %)
Income above NOK 300’	
No	641 (56 %)
Yes	379 (33 %)
Not answered	125 (11 %)
Cooperation agreement on a more inclusive working life	
No or do not know	285 (25 %)
Yes	635 (55 %)
Not answered	225 (20 %)
Working ability score when entering rehab	
Score 1-3 (high to medium ability)	435 (38 %)
Score 4	438 (38 %)
Score 5 (low ability)	272 (24 %)
Diagnosis group at baseline	
Musculoskeletal	682 (60 %)
Mental	232 (20 %)
Other	231 (20 %)

The transition intensities for the 15 transitions in the multi-state model from Fig. 1 were examined using the Nelson-Aalen estimator for marginal transition intensities, and Cox proportional hazards and Aalen additive hazards models for conditional transition intensities using relevant covariate information. Cox and Aalen models were fitted using either the coxph or aareg function in the survival package [36] of the statistical software R [28]. The Nelson-Aalen estimator was calculated by using the coxph function without covariates.

Say that *X*(*t*) denotes the state for an individual at time *t*. The transition probability matrix ***P***(*s*,*t*), with elements *P*_*hj*_(*s*,*t*)=*P*(*X*(*t*)=*j*∣*X*(*s*)=*h*), denoting the transition probability from state *h* to state *j* in the time interval (*s*,*t*], was then estimated by the matrix product-integral formula

(1) $$ \hat{\boldsymbol{P}}(s,t) = \prod_{u \in (s,t]} \big(\boldsymbol{I} + d\boldsymbol{\hat{{A}}}(u)\big),   $$

where $\boldsymbol {\hat {{A}}}(u)$ is the corresponding estimated cumulative transition intensity matrix at time *u* [13, 29]. The cumulative intensities in $\boldsymbol {\hat {{A}}}(u)$ are estimated using the Nelson-Aalen estimator.

The cumulative transition intensity matrix could also be estimated conditioning on covariates *Z*, changing the formula in Eq. 1 to

(2) $$ \hat{\boldsymbol{P}}_{Z}(s,t) = \prod_{u \in (s,t]} \big(\boldsymbol{I} + d\boldsymbol{\hat{{A}}}_{Z}(u)\big),   $$

where $\boldsymbol {\hat {P}}_{Z}(s,t)$ and $\boldsymbol {\hat {A}}_{Z}(u)$ are the estimated covariate specific transition probability matrix and cumulative transition intensity matrix respectively. The cumulative intensities in $\boldsymbol {\hat {A}}_{Z}(u)$ is estimated for given values of *Z* using Cox proportional hazards models or Aalen additive hazards models.

From the estimated transition probability matrix one can study the probabilities of being in state *j* at time *t* when starting in state *h* at baseline, $\hat {P}_{\textit {hj}}(0,t)$, or the overall probability of being in state *j* at time *t*,

(3) $$ \hat{P}(X(t) = j) = \sum_{k} \hat{P}_{kj}(0,t) \cdot \hat{P}\big(X(0)=k\big).   $$

For models without covariates, *P*(*X*(0)=*k*) can be estimated by the proportion starting in state *k*. With covariates, it can be estimated using logistic regression.

With cumulative hazard estimates from the Nelson-Aalen estimator, the formula in 1 corresponds to the Aalen-Johansen estimator. With this marginal approach or with covariate adjusted cumulative hazards like in Eq. 2 estimated using Cox proportional hazards models, estimates and confidence intervals were calculated using the mstate package [29]. Using cumulative hazard estimates from Aalen additive hazards models, the estimator from Eq. 2 has to be implemented separately. Confidence intervals can be calculated using bootstrap methods or analytically as described in Aalen, Borgan and Gjessing ([37], p. 183).

Note that there is an intrinsic Markov assumption [13] in this way of multi-state modelling which can be challenging when using complex data such as data based on sick leave and disability benefits. When the length of stay in a state affects the intensity for leaving the state, this assumption is in principal being violated. This is the case in three of the states in our multi-state model due to administrative regulations. Individuals can only be on sick leave or partial sick leave spells of maximum one year, and on work assessment allowance for a maximum of four years. To what degree such violations pose a problem will however depend on how often individuals stay in these states long enough for the regulations to take effect, which again partly depend on the follow-up time of the study. In our study we have individual follow-up times ranging between three and five year, which means that the maximum time of four years for work assessment allowance not will pose a problem. In fact, the mean length of stay in this state is 274 days (with a 95 % percentile of 1028 days). Also sick leave and partial sick leave spells close to a year is very rare in our study population, with a mean stay of 38 days on sick leave and 68 days on partial sick leave (and corresponding 95 % percentils of 180 and 218 days). Overall, this seems to indicate that while serious violations to the Markov assumption are possible, they are in practice uncommon and should not make any big impacts on the results for our study. However, in general one should be aware that violations of this assumption may impact some of the estimated effects, including the causal parameters of interest.

Note also that more advance models relaxing the Markov assumption have been developed, but the impact of such violations will vary and could often be disregarded. See for example Gunnes et al. [38] and Allignol et al. [39], who only show small discrepancies between Markov and non-Markov models in situations where the Markov assumption is not met. When focusing on overall state occupation probabilities as in Eq. 3, Datta and Satten [40] have showed that the product-integral estimator in Eq. 1 is consistent regardless of whether the Markov assumption is being valid.

### Causal inference and the effect of interventions in multi-state models

Besides estimating transition intensities and probabilities for a given set of states in a multi-state model and doing individual predictions, it is also of interest to evaluate population average effects of interventions in the multi-state model framework. There is a fundamental difference between merely predicting covariate specific outcomes and to estimate the causal effect of intervention on them, which creates a need for special methods and assumptions. We now consider three different approaches based on classical methods from the causal inference literature.

The methods are exemplified with regard to the two types of possible interventions mentioned in the Introduction. The first intervention is the use of partial versus full time sick leave, where partial sick leave often is thought to cause shorter absence and higher subsequent employment [14]. The other intervention is the use of cooperation agreements on more inclusive working life, which in Norway has been implemented with the goal of improving work environment, enhance presence at work, prevent and reduce sick leave and prevent exclusion and withdrawal from working life. A secondary aim is to prevent withdrawal and to increase employment of people with impaired functional ability. Participating enterprises must systematically carry out health and safety measures, with inclusive working life as an integral part, and will in return receive prevention and facilitation subsidies and have their own contact person at NAV [41]. Note that the first of these two interventions is represented through states in our multi-state model in Fig. 1, while the latter is represented as an additional covariate as shown in Table 2.

As for causal assumptions we will focus on the three general conditions which have been identified for estimating average causal effects; positivity, exchangeability (“no unmeasured confounding”) and consistency (“well-defined interventions”) [42]. We will also discuss how the related modularity condition, e.g. from the Pearl framework of causal inference [26], is relevant in our context of multi-state models. Additionally, as always, we need the statistical assumptions of no model-misclassification, which in our case is important both at an intensity and overall multi-state level. The importance and validity of all these assumptions are discussed separately for the three different approaches in following sub sections.

#### Artificially manipulating transition intensities

One proposed method for making causal inference in multi-state models is to artificially change certain transition intensities in $\boldsymbol {\hat {{A}}}(u)$ and then explore the corresponding hypothetical transition probabilities [43]. Such changes in transition intensities, creating a new transition intensity matrix which can be denoted $\boldsymbol {\tilde {{A}}}(u)$, may represent interventions. The hypothetical transition probabilities, which we can denote $\boldsymbol {\tilde {{P}}}(s,t)$, may then represent counterfactual outcomes. Confidence intervals for such hypothetical transition probabilities can be found through the distribution of the cumulative intensities after manipulation. For situations without covariates and for the additive hazards model this will follow by the arguments in Aalen, Borgan and Gjessing ([37], p. 123–126 and 181–190). For the Cox model it will follow by the functional delta method in Andersen, Borgan, Gill & Keiding ([44], p. 512–515). For more on these types of analyses with respect to causal inference, and especially the connection to G-computation, see Keiding et al. [43] and Aalen, Borgan and Gjessing ([37], p. 382).

The important causal assumption for this approach to be reasonable is that when intervening on a set of transition intensities, the remaining transition intensities stay unchanged. This is equivalent to the modularity assumption and definition of a structural causal model in the Pearl framework of causal inference [26]. See Aalen et al. [45] and Røysland [46] for more on modularity in the light of intensity processes. However, even when it is unreasonable that such an assumption is fully met, it has been argued that this kind of inference in multi-state models still can give valuable insights ([47], p. 250).

In this paper we will follow the ideas from Keiding et al. [43] for our multi-state model for sickness absence and work in Fig. 1, and define interventions through manipulating transition rates within given sets of covariate values, where such interventions would be realistic. One example of an intervention would be to increase the use of partial sick leave compared to full sick leave, which would correspond to modifying the intensities into the partial sick leave and sick leave states.

For the modularity assumption to be met in this case, it means that the additional individuals counterfactually put on partial sick leave instead of full sick leave, should behave identical to those individuals who were observed on partial sick leave in the original data. As those on partial sick leave generally are in a better health state than those on full sick leave, this is not a reasonable assumption. However, it is reasonable within similar stratums of covariate levels, which we will study in later in this paper. Satisfying the condition of modularity in this manner, also will imply that the assumptions of positivity, exchangeability and consistency are met.

#### Inverse probability weighting

Another approach from the causal inference literature is inverse probability of treatment (or propensity score) weighting [48, 49]. The treatment or exposure of interest can be represented either as states in the multi-state model or through additional covariates. One could for example weight by the inverse probability of being in a given state at baseline, before estimating the transition intensities of the model in Fig. 1. This would correspond to modelling a counterfactual scenario where there is a copy of each individual in every possible initial state.

The sufficient conditions for this approach to be valid is again the causal assumptions of positivity, exchangeability and consistency. Positivity here means that there should be a non-zero probability of receiving all possible exposures for all covariate values in the population. Also, the model for the exposure, which is the foundation for the weights, must be well specified. See for example [42, 48, 49] for a further discussion on these assumptions.

Say that we would like to compare the effect of being put on sick leave versus partial sick leave at baseline (when being discharged from the rehabilitation center). Let us for now only consider those starting in either of these two states. Whether an individual is put on full or partial sick leave at baseline is hardly randomized. We could however model the counterfactual situation where everyone, regardless of their covariate information, were put on full sick leave at baseline and an identical copy of each individual were placed on part time sick leave. This can be achieved by applying the weights

$$ w_{k} = \frac{1}{P(S_{k} = s_{k}|Z_{k} = z_{k})},     $$

where *S*_*k*_ is the initial state and *Z*_*k*_ is all the relevant covariate information explaining the initial state for individual *k*. The probabilities of being in either of the two states at baseline can be estimated using ordinary logistic regression. The uncertainty of the estimates from the resulting weighted multi-state analysis can easily be calculated using for example the coxph function in R with robust standard errors [50].

Another casual contrast of interest would be to compare the scenario where everyone got a cooperation agreement on a more inclusive working life with a scenario where no-one had such an agreement. This would correspond to modelling a situation where such agreements were randomized. This could be modelled by weighting every individual in the original data with the inverse probability of having a cooperation agreement on a more including working life given covariates, by applying the weights

$$ w_{k} = \frac{1}{P(E_{k} = e_{k}|Z_{k} = z_{k})},     $$

where *E*_*k*_ is an indicator variable that is 1 if an agreement is present and 0 otherwise. The probabilities can again be estimated using logistic regression.

Assuming positivity for the first type of intervention means that there should be a probability greater than zero for starting in either of the two states of sick leave or partial sick leave at baseline, regardless of any observed covariate history. This is testable, and the covariates in Table 2 are well balanced over the two groups. The biggest difference lies in the distribution of the working ability score, but even in the partial sick leave group 5 % of the individuals has a low ability score (the lowest health score). As for exchangeability it is a question of whether the included covariates sufficiently explain the differences between those on full and partial sick leave at baseline. The covariates include demographic, socioeconomic, work and health variables, which should be the central parameters. However, to what degree they are sufficiently covered is untestable. The health variable should ideally had been collected at baseline, and not at the first measurement after entering the rehab, but one could hope that in combination with type of work and diagnosis group, it will still be sufficient. An example of a variable that was considered, but not included, is the center that the patients attended. Adding this information, which involves adding 7 new dummy variables, seemed to have little impact. We therefore assume that center specific differences between patients are covered sufficiently through the other covariates, and especially working ability score and diagnosis group. For the cooperation agreement intervention, this is not administered at an individual level, and thus the assumptions are even easier to assess. There are no covariate combinations that exclude such agreements and the most important confounder will be type of work. Both interventions can also be assumed well-specified.

#### G-computation

A third approach, which corresponds to G-computation [51–53] (or standardization) of the parameter from the inverse probability weighting, is to estimate the transition intensities for individual *k* conditioned on all relevant covariate information *Z*_*k*_ using a Cox proportional hazards or an Aalen additive hazards model, and then predict the state transition probabilities given covariates *Z*, *P*_*hj*_(*s*,*t*∣*Z*), for every individual given a specific intervention. As for the inverse probability weighting approach, the intervention could be defined both through setting a specific initial state or a covariate to a specific value.

The main causal assumptions are again positivity, exchangeability and consistency, together with the assumption of no model misspecification. However, the model which needs to be correctly specified is now the model for the outcome, and not a model for the exposure as for the inverse probability approach. See for example [52] for a discussion on the causal assumptions of G-computation. For a general discussion on the use of inverse probability weighting and G-computation, and the connection to standardisation, see [53].

If, again, we would like to compare the effect of being put on sick leave versus partial sick leave at baseline, the intervention would correspond to setting their initial state to *h*=2 and *h*=3, and compare all individual predictions for both values. The population average effect can then be estimated through

$$ \frac{1}{n} \sum_{k} \hat{P}_{3,j}(0,t \mid Z_{k}) - \frac{1}{n} \sum_{k} \hat{P}_{2,j}(0,t \mid Z_{k}),    $$

where *n* is the number of individuals in the study. Confidence intervals can be found using standard bootstrap techniques.

Correspondingly, if we consider an intervention such as the cooperation agreement on a more inclusive working life, represented by a binary covariate *E*_*k*_, the population average effect of such an intervention can be estimate by

(4) $$ \frac{1}{n} \sum_{k} \hat{P}_{i,j}\left(0,t \mid Z_{k}^{E_{k}=1}\right) - \frac{1}{n} \sum_{k} \hat{P}_{i,j}\left(0,t \mid Z_{k}^{E_{k}=0}\right),   $$

for given initial states *i*.

As these interventions are the same as the ones in question for the inverse probability approach, the causal assumptions need are also identical. See the discussion of these assumptions in the previous sub section.

## Results

### Unadjusted analysis

**Fig. 2:**
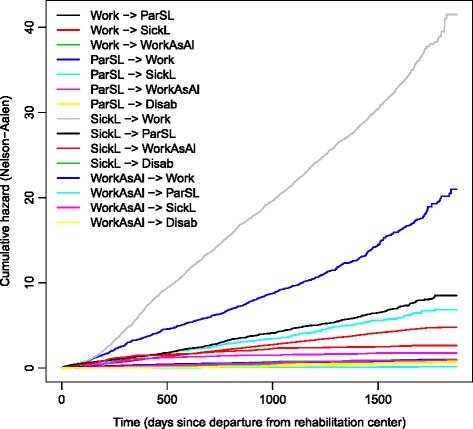
Nelson-Aalen estimates of unadjusted cumulative transition intensities for the 15 transitions in the multi-state model. The five states in the model is work (Work), sick leave benefits (SickL), partial sick leave benefits (ParSL), work assessment allowance (WorkAsAl) and disability pension (Disab)

Unadjusted cumulative intensities for the 15 transitions in the multi-state model in Fig. 1 estimated using the Nelson-Aalen estimator are found in Fig. 2. We see how the magnitude of the estimated transition intensities varies between states, and that transitions from sick leave to work has the highest intensity. Note that estimated intensities will correspond to the slopes of the cumulative estimates in this figure.

**Fig. 3:**
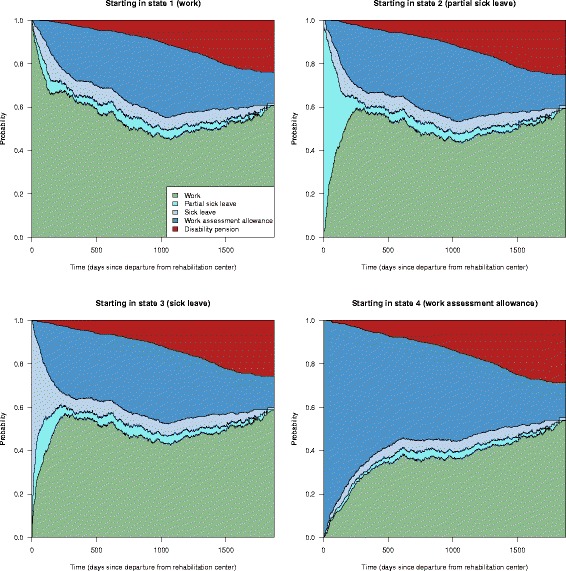
Unadjusted state transition probabilities. Predictions given the patients state at baseline (time of discharge from the work rehabilitation center)

The estimated time-varying transition probabilities, found by Eq. 1, give rise to the stacked probability plots in Fig. 3, given the four possible initial states (work, sick leave, partial sick leave and work assessment allowance). For example, we see that an individual who is on sick leave at time 0, has an unadjusted probability of approximately 0.50 of having returned to work after three years. The unadjusted probability of being disabled after the same period is approximately 0.10.

**Fig. 4:**
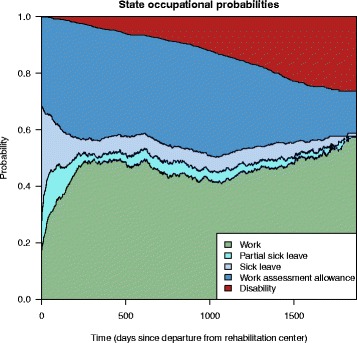
State occupation probabilities. The overall probability of being in the five states over time, estimated using Eq. 3

Overall state occupation probabilities calculated according to (3) are shown in Fig. 4. We see that, for example, overall there is a rapid increase in work after being discharged from the rehabilitation center, from just below 20 % to just below 50 % after the first year. The general tendencies in this figure are similar to the ones in the paper by Øyeflaten et al. [3], who do an unadjusted analysis on a subset of the patients included in the current analysis. Note that in the remainder of this paper we focus on state transition probability plots, but that similar plots of the state occupation probabilities also can be derived.

### Covariate adjusted analysis and individual predictions

**Fig. 5:**
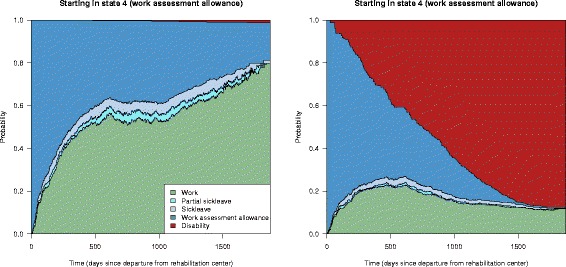
Covariate adjusted state transition probabilities with work assessment allowance as the initial state; predictions given two selected sets of covariates. *Left panel*: Married female aged 30 in an educational job, with a cooperation agreement on a more inclusive working life, income above NOK 300 000, higher education, working ability score 4 and mental diagnosis. *Right panel*: Single male aged 60 in a manual job, no cooperation agreement on a more inclusive working life, income below NOK 300 000, no higher education, work ability score 4 and musculoskeletal diagnosis

Adjusting for the covariates age, gender, marital status, higher education, type of work, income, cooperation agreement on a more inclusive working life, work ability score and baseline diagnosis when estimating the transition hazards, allows for covariate specific predictions of the state transition probabilities. Figure 5 shows two examples of such predictions, for a married female aged 30 in an educational job, with an agreement on inclusive working life, income above NOK 300 000, higher education, working ability score 4 and mental diagnosis, and a single male aged 60 in a manual job, no agreement on inclusive working life, income below NOK 300 000, no higher education, work ability score 4 and musculoskeletal diagnosis. Note that when fitting the models, from the original covariates described in Table 2, those who did not answer the questions on marital status, higher education or having an inclusive working life agreement were put in the “no” category. We see that the estimated state transition probabilities for the two sets of covariates clearly differ with respect to work. The probability of returning to work within the follow-up time is almost 0.80 for females with the given example of covariates, while only about 0.10–0.15 for males in the second example.

**Fig. 6:**
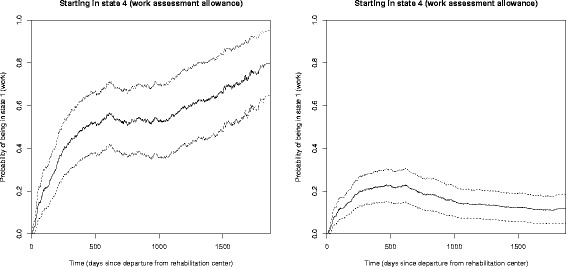
The probability of being in state 1 (work) after starting in state 4 (work assessment allowance) for two covariate specific predictions. *Left panel*: Married female aged 30 in a educational job, with a cooperation agreement on an inclusive working life, income above NOK 300 000, higher education, working ability score 4 and mental diagnosis. *Right panel*: Single male aged 60 in a manual job, no agreement on a more inclusive working life, income below NOK 300 000, no higher education, work ability score 4 and musculoskeletal diagnosis

Note that the stacked probability plots in Figs. 3 and 5 do not include confidence intervals. In Fig. 6 we explore these by showing the probability of having returned to work from state 4 (work assessment allowance) at any time, with corresponding confidence intervals, for the two scenarios in Fig. 5. We see that the probability of returning to work after being on work assessment allowance is very different for individuals with the two different sets of covariates, also when accounting for the uncertainty of the estimates.

The results using a Cox proportional hazards model were also compared with an Aalen additive hazards model for modelling the transition intensities in our multi-state model. Even in simple additive models where constant hazards were assumed, we saw a good agreement between additive and proportional hazards models. See the next sub section for a further comparison between these two types of hazard models.

### The effect of hypothetical interventions

**Fig. 7:**
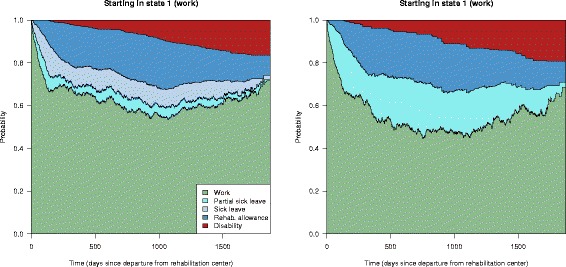
Results intervening on sick leave intensities. Comparing predicted *(left panel)* and counterfactual *(right panel)* state transition probabilities for a selected set of covariates: Married male aged 45 in a service job, with no agreement on a more inclusive working life, income below NOK 300 000, no higher education, with a high to medium working ability score and musculoskeletal diagnosis. In the counterfactual scenario all transitions into sick leave have been blocked and routed into partial sick leave

Let us now consider results from the three proposed methods for doing causal inference in our multi-state model. For assessing hypothetical interventions on the use of full and partial sick leave benefits in the multi-state model in Fig. 1, let us first look at a scenario where we artificially manipulate the transition intensities that go into the partial sick leave and sick leave states. Figure 7 show the state transition probabilities for an individual starting in the work state at baseline. The left panel show the estimated probabilities given the original multi-state model, while the right panel show a counterfactual scenario where all transitions into full sick leave are blocked and routed into partial sick leave. This manipulation of the multi-state model corresponds to removing the possibility of full time sick leave, and instead putting individuals on partial sick leave. For such a manipulation to be reasonable, this should be done within a set of covariate characteristics where this intervention is realistic. The figure shows results for married males aged 45 in an educational job, income below NOK 300 000, no higher education, working capacity score 1 and a musculoskeletal diagnosis. From Fig. 7, we see that the state transition probabilities are similar for the two scenarios, but that individuals tend to quit full time work more frequently when full time sick leave is not available. The use of part time sick leave benefits is of course higher, but the use of work assessment allowance and disability pension is actually lower.

**Fig. 8:**
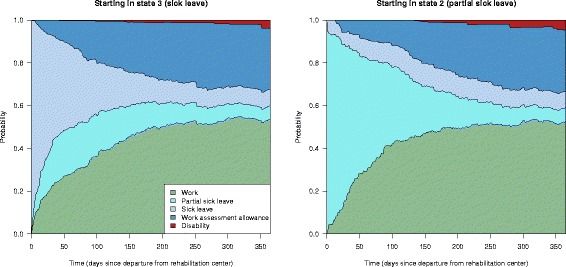
Inverse probability weighting results for full versus partial sick leave. State transition probabilities for the counterfactual scenarios where everyone originally on full or partial sick leave were given full sick leave *(left panel)* or partial sick leave *(right panel)*. Note that time axis is restricted to the first year, to highlight the differences between these two scenarios

Let us then consider the inverse probability weighting approach and first the hypothetical intervention of placing all individuals on either full or part time sick leave at baseline. To assess such an intervention we focus only on the individuals in partial or full sick leave at baseline and give them a weight corresponding to the inverse probability of starting in their initial state. Then we estimate all transition intensities of the multi-state model in Fig. 1 and calculate their state transition probabilities as functions of time. This will correspond to comparing partial and full sick leave as if it was randomized at baseline. State transition probabilities for these two scenarios are shown in Fig. 8. Note that when intervening on initial states in a model that is Markov, like we do here, the differences between the two interventions will be smaller and smaller with time. When comparing partial and full sick leave, the difference is mostly visible during the first year. To give a more detailed picture of this difference, the time axis in Fig. 8 has been restricted to go from 0 to 365 days. Probabilities of starting in a given initial state were calculated using logistic regression, adjusting for the covariates in Table 2. We see that there is a tendency that partial sick leave yields a faster return to work than full time sick leave, and to a certain degree replace the use of work assessment allowance, but the differences are small.

**Fig. 9:**
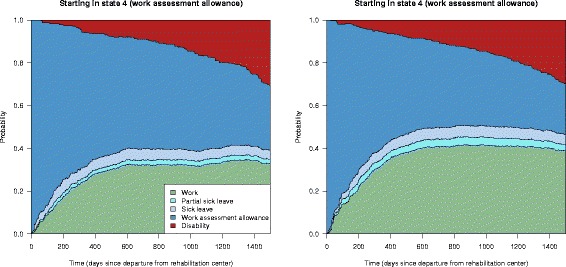
Inverse probability weighting results for the effect of having a cooperation agreement. State transition probabilities for the counterfactual scenarios no-one has a cooperation agreement on a more inclusive working life *(left panel)* and the scenario where everyone has such an agreement *(right panel)*

Another intervention in question was the cooperation agreement on a more inclusive working life. The effect of this agreement could be assessed by weighting with the inverse probability of having an agreement and then look at the transition probabilities for the weighted subsets of the original data for those without and with an agreement. This corresponds to modelling two counterfactual scenarios; one where no-one has such an agreement and another where everyone has one. The results from such a comparison is shown in Fig. 9. Probabilities of having an agreement were calculated using logistic regression, adjusting for the covariates in Table 2. We see that there is a small but positive effect of having an agreement on a more inclusive working life with respect to having a higher probability of returning to work.

**Fig. 10:**
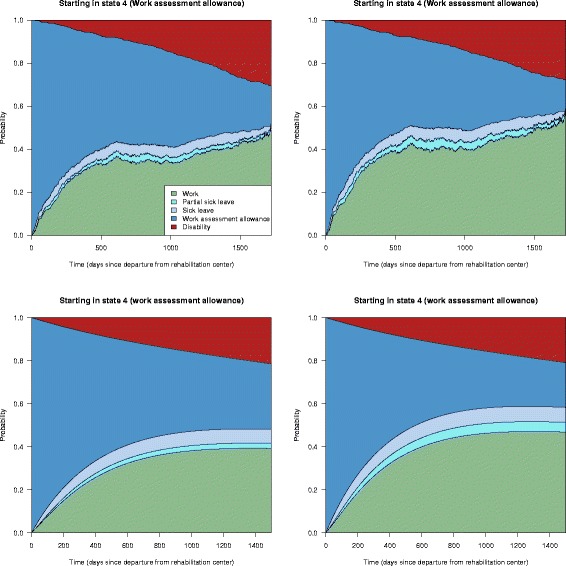
G-computation results for the effect having a cooperation agreement. State transition probabilities for the counterfactual scenarios no-one has a cooperation agreement on a more inclusive working life *(left panels)* and the scenario where everyone has such an agreement (right panels), estimated using the G-computation approach with Cox proportional hazards models *(upper panels)* and Aalen additive hazards models *(lower panels)*

Finally, if we consider the G-computation approach, we can again estimate the effect of having an agreement on a more inclusive working life by estimating state transition probabilities for every individual when the indicator variable for such an agreement first is fixed to 0 and then 1, and look at average predictions for all individuals. The average predictions can be seen in Fig. 10, from using a Cox model in the upper panels and from an Aalen additive model in the lower panels. The two hazard models give very similar results. The smooth curves for the additive models is due to the assumption of constant hazard rates, which simplifies the model fitting. Left panels show overall state transition probabilities without an agreement and the right panels show overall transition probabilities with an agreement. We again see a small but positive effect of having such an agreement. We also see that the results are very similar to the results when using the inverse probability weighting approach in Fig. 9. As described earlier, a similar analysis can be done with regards to starting in partial or full time sick leave at baseline. Again, results (not shown) are similar to the ones estimated using inverse probability weights (shown in Fig. 8).

**Fig. 11:**
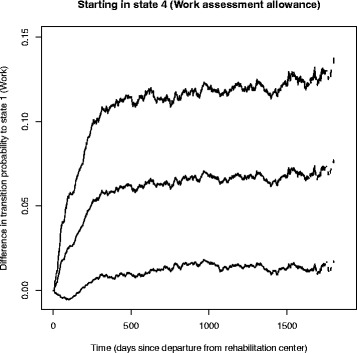
Effect of having a cooperation agreement. Difference in probability of returning to work for the two counterfactual scenarios where no-one has an agreement on more inclusive working life and the scenario where everyone has such an agreement, estimated using the G-computation approach. Ninety-five percent bootstrap confidence intervals are presented around the effect

An alternative way to illustrate the effect of the inclusive working life agreement is to plot the difference in state transition probabilities, for instance of returning to the work state from work assessment allowance. Ninety-five percent confidence intervals for such effects can be found using bootstrap techniques. Note however that such a bootstrap can be computationally heavy, for example in the G-computation approach when averaging over all individual predictions. A possible shortcut is however to make one prediction for average covariate levels together with the manipulated covariate. Formally, this can be justified for additive hazards models, but in our applications we found that it also gave a good approximation with Cox models. Results from such an analysis can be found in Fig. 11, using Cox proportional hazards models to estimate the causal effect in Eq. 4, and the latter bootstrap approach for confidence intervals. We see that, after the first year, there is a rather constant positive effect of having a cooperation agreement on a more inclusive working life, with about 5 percent points higher probability of entering the work state. However, the uncertainty is relatively high, with a 95 % bootstrap confidence interval ranging from about 1 percent to 10 percent.

## Discussion

One of the important goals of sickness absence research is to find effective interventions for controlling it. Registry data on sickness benefits is a primary source for making such inference, and multi-state models have proved to be a very successful framework for modelling the transitions between different benefits and work in such data. Coupling registry data with detailed information about cohort participants, gives further insights about underlying reasons for sickness absence and can predict patient specific probabilities of future sickness absence, disability and returning to work. Combining these methods with standard methods from causal inference is a first attempt to then answer questions of the effect of interventions. In this paper we have considered examples of two such possible interventions; namely the use of partial sick leave and cooperation agreements for a more inclusive working life.

Covariate specific predictions show great differences in the probabilities for sick leave, disability and work for patients with assumed high risk and low risk covariate characteristics. Overall, we find small effects of partial sick leave compared to full sick leave on state transition probabilities. Note however that in terms of expenses, partial sick leave benefits are less costly than giving full sick leave benefits, and thus, no difference in outcome between the two would indicate that partial sick leave should be preferred when possible. For cooperation agreements on a more including working life we find more visible, but still rather small, effects. Again, in terms of overall expenses, the effects of having such agreements must be considered against the cost of implementing them.

When it comes to graphically representing the outcome in multi-state models there are many possibilities, and we have only looked at some of them. Stacked probability plots are illustrative, of either state transition or state occupation probabilities, while non-stacked plots make it easier to include confidence intervals. When assessing the effect of interventions one can plot the difference in these probabilities, as we have done, or alternatively the ratio between state transition or occupation probabilities. Another possible outcome measure could be to study the area under each curve, which will correspond to the expected time spent in each state during follow-up.

Methodologically, the graphical features of the multi-state model framework makes it very suitable for thinking in terms of causal inference. Both in terms of the intuitiveness of defining interventions in terms of manipulating transition intensities, but also in terms of interpreting the outcomes of interventions using state transition and occupation probabilities. We also find that standard approaches from the causal inference literature, such as inverse probability weighting and G-computation, can help identify causal parameters easily interpreted also in a multi-state model setting. The methods applied in this paper are kept rather simple, partly for illustrative purposes, but can also easily be extended to estimate effects of time-varying exposures or interventions and to compare treatment regimes. One should however expect that this makes both standard model assumptions and causal assumptions harder to meet.

For the modelling of transition intensities it is reassuring that the Cox proportional hazards models and Aalen additive hazards models gave similar results. The two models have different advantages in the setting of this paper. The Cox model is easier to implement using existing software, while the additive model needs more model fitting assessment, for example in deciding how to smooth the estimated cumulative hazards to get well behaved hazard estimates. In this paper we could assume constant intensities for the additive hazards models which simplifies the model fitting. When doing individual predictions, the additive models are not ideal, as they can give probability estimates below 0 or above 1 for uncommon combinations of covariates. A major benefit of the additive hazards model however, is that because of their additive structure, predicting with average covariate values is a shortcut to the individual predictions used in the G-computation approach. Apart from the standard model assessments when fitting separate hazard models for each transition, the most important statistical assumption to consider is of course the Markov assumption for the overall multi-state model, which was discussed in the Methods section.

As for causal assumptions, it is clear that with the complexity of multi-state models, causal interpretation should not be made naively. To interpret all the separate transition intensity models and the overall multi-state model causally is challenging. To what degree such causal assumptions are needed will however depend on the approach used to define the intervention of interest. When intervening on transition intensities, the structural assumption of the full model will be key, while when intervening on treatment indicator variables, such as in the approach referred to as G-computation, the causal interpretation of the coefficient for this variable, in each separate hazard model, will be of particular importance.

The goal of this paper, in terms of causal inference, is to illustrate how standard approaches can be used in a multi-state model setting to answer questions about the effect of interventions. When it comes to formal arguments for the validity of these approaches there is room for more work, especially on the sensitivity of the Markov assumption and how deviation will affect the validity of the causal assumptions. Overall, we believe that there are many benefits from thinking in terms of causal inference for multi-state models, as research questions often boil down to questions on the effect of interventions. It is also worth noticing that many of these approaches have been used at some level in multi-state models also historically. In particular, this goes for manipulating transition intensities and fixing covariate values, which in this paper was put in a G-computation context. However, few formal connections have yet been made to the causal inference field.

## Conclusions

Detailed covariate information is important for explaining transitions between different states of sickness absence and work in a multi-state model, also for patient specific cohorts. Methods from the causal inference literature can provide the needed tools for going from covariate specific estimates to population average effects in in such models, and thus yield new insights when assessing hypothetical interventions from complex observational data.
